# Supplementary material for: Ageing and the Ipsilateral M1 BOLD Response: A Connectivity Study
Source: Brain Sci. 2021 Aug 26;11(9):1130. doi: 10.3390/brainsci11091130 (PMC8470146; doi:10.3390/brainsci11091130)
Supplement: Supplementary file 1 [file brainsci-11-01130-s001.zip › brainsci-1337069-supplementary.pdf]

Supplementary material

Ageing and the ipsilateral M1 BOLD response: a connectivity study

Yae Won Tak, Ethan Knights, Richard Henson and Peter Zeidman

1. Supplementary Methods

1.1 Preparation of behavioural covariates

We included the 17 covariates for our SPM analyses, listed in Table S1. The age regressor was mean-centred before calculating the higher order terms, and the three age terms were orthogonalised using recursive Gram-Schmidt orthogonalisation (with the Matlab function spm_orth.m).

Table S1. Covariates used in the behavioural analysis and their definition.

	**Variable**	**Cam-CAN home interview code**
1	Age	
2	Age squared	
3	Age cubed	
4	Sex	
5	Handedness [A]	
6	MR coil	
7	Smoking [B]	v241, v252, v259
8	Blood pressure	v349
9	Cholesterol	v355
10	Cardiac [C]	v357, v359, v361, v370, v372
11	Migraine	v366
12	Diabetes	v377
13	Height	
14	Weight	
15	Blood pressure (systolic) [D]	
16	Blood pressure (diastolic) [D]	
17	Resting pulse rate [D]	
18	Hearing loss	v336, v342
19	Hearing test score (left) [E]	v346
20	Hearing test score (right) [E]	v344
21	Hearing test unavailable	v344, v346

(A) Edinburgh handedness inventory (EHI). (B) Whether the subject smokes now, or had previously smoked on at least 100 occasions, or if anyone in their household smokes. (C) Any one of: angina, heart attack, cardiac arrhythmia, pulmonary embolism, deep vein thrombosis. (D) Resting values averaged over the second and third of three measurements per subject. (E) Number of 1Khz tones heard (max 3).

1.2 SPM analysis

For each subject we specified a general linear model (GLM) with three conditions of interest: auditory+visual trials (collapsed over auditory frequency), auditory only trials and visual only trials. Each condition was modelled as a separate box-car regressor with trials of duration 300ms, which were convolved with the default canonical haemodynamic response function (HRF) in SPM. We also included six regressors encoding head movement and a regressor to model the session mean.

To test for commonalities across subjects and differences due to age, we ran one-sample t-tests across subjects using SPM, separately for the auditory+visual, auditory and visual conditions. The variables in Table S1 were included as covariates. For each of the three models, we computed an F-contrast to test for any effect of age (specified as an identity matrix of dimension three across age, age squared and age cubed, to capture both linear and non-linear effects). Results were rendered onto 3D cortical surfaces using BrainNet Viewer (http://www.nitrc.org/projects/bnv/) [1].

2. Supplementary Results

2.1 SPM: average response to auditory+visual trials

We first identified regions of the brain responding to the task, averaged across all subjects (Figure S1). Most voxels showed task-related effects, which is unsurprisingly given the large number of subjects and the use of classical statistics. As expected, a particular difference can be seen between hemispheres in sensorimotor cortex, in which the right hemisphere (circled) has negative responses, whereas the left hemispheres has positive responses.

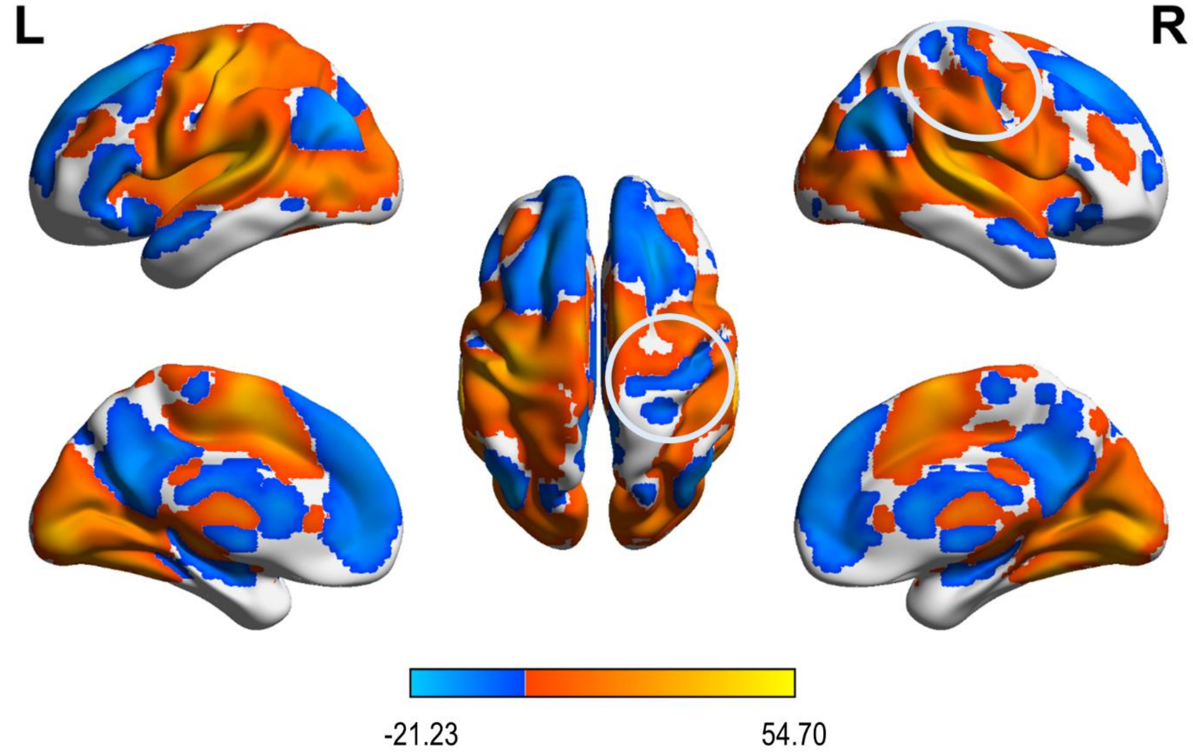

Figure S1. The main effect of task (auditory+visual trials). Results of a T-contrast are projected onto the ICBM152 (smoothed) template, thresholded at P < 0.05 family-wise error corrected. Hot colours indicate positive responses (relative to unmodelled time, particularly inter-trial intervals) and cold colours indicate negative responses. The circles highlight negative BOLD in right sensorimotor cortex.

2.2 SPM: effects of age

We next identified locations in the brain showing systematic effects of age. We fitted a second level regression model for auditory+visual trials, including age effects as covariates while controlling for other variables that could affect neurovascular coupling (listed in Table S1). An F-contrast for effects of age, covering linear, quadratic and third order terms, identified significant clusters including right sensorimotor cortex, bilateral supplementary motor area (SMA) and bilateral primary auditory cortex (Figure S2 and Table S2). Post-hoc t-tests (not shown) demonstrated that the age effects in bilateral SMA and right sensorimotor cortex were positive (i.e., more positive activity in older people) whereas effects in primary auditory cortex were negative (i.e., less positive activity in older people).

We used an atlas called the Human Motor Area Template (HMAT) [2] to label the regions that constituted the right sensorimotor cluster (arrows in Figure S2). 60% of voxels were in right M1, 23% were in right S1 and 12% were right dorsal premotor cortex. The remaining 5% were outside the atlas.

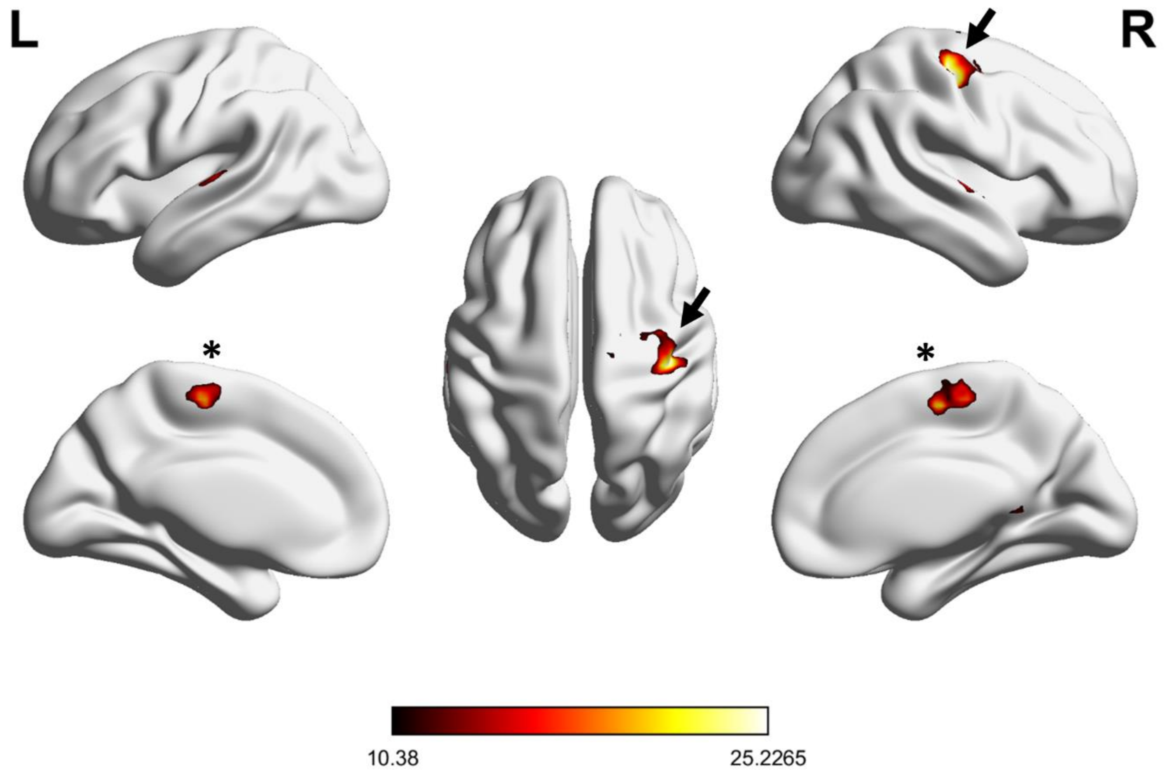

Figure S2. Effects of age on the response to auditory+visual trials. Results of an F-contrast are projected onto the ICBM152 (smoothed) template, thresholded at P < 0.05 family-wise error corrected. Arrows indicate a cluster that includes right primary sensory cortex, right primary motor cortex (M1) and right dorsal premotor cortex (dPM). Asterisks indicate bilateral supplementary motor area (SMA). The colour bar indicates the F-statistic.

Table S2. Effects of age on whole-brain fMRI results

**Description**	**Peak XYZ (MNI, mm)**	**Voxels***	**Peak F**
Right precentral gyrus / postcentral gyrus (M1)	39 -21 54	293	27.3
Bilateral SMA	6 -12 54	124	21.8
Left planum temporale / Heschl's gyrus	-57 -24 6	89	17.2
Right planum polare / central opercular cortex / temporal pole	48 3 -3	73	19.7
Left cerebellum (V,V1)	-18 -48 -21	21	16
Right brain stem	3 -42 -36	18	20.1
Right precentral gyrus / superior frontal gyrus	15 -15 69	17	13.2
Right retrosplenial cortex	9 -48 6	14	15.6
Right precentral / postcentral gyrus	12 -30 69	14	16.4
Right cerebellum (V, I-IV)	15 -45 -18	8	12.3

Left postcentral / precentral gyrus	-9 -33 69	8	14.9
Right thalamus	9 -21 -3	6	12.6
Left precuneus	-6 -63 24	6	13.2
Left angular gyrus / lateral occipital cortex	-48 -57 27	5	12.7

* Table truncated for brevity: only clusters with five or more voxels reported.

2.3 ROI analyses

We defined eight regions of interest, as detailed in the main text. To reprise, the lSMA, rSMA, rPMd and rM1 were identified in the SPM analysis on the basis of having a non-linear (3rd order) mapping between response and age. The remaining two regions, lPMd and lM1, were homologues of their right hemisphere counterparts. Figure S3 shows the voxels included in the ROIs of all subjects.

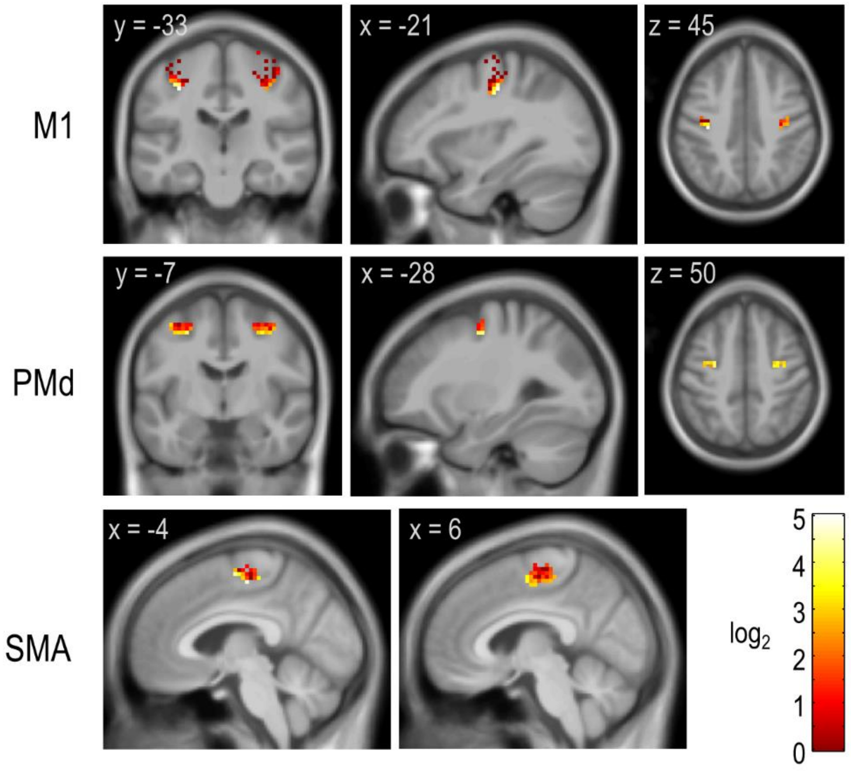

Figure S3. Voxels that were used to generate a representative timeseries for each region of interest (ROI). The colour bar indicates the (log) number of subjects for whom each voxel was included. Data ranged from 1 subject (log_2 1=0) to 131 subjects (log_2 131=4.88) for each voxel. MNI coordinates are shown inset and in the background are slices of the group average structural image.

We extracted first-level GLM parameters corresponding to the Auditory+Visual condition, averaged over voxels within each ROI. To confirm that the ROI-averaged results qualitatively matched the voxel-wise results, we calculated (post-hoc) linear correlations between response and age, which unsurprisingly were significant in lSMA, rSMA, rdPM and rM1. To illustrate the non-linear association between response and age, we fitted 3rd order polynomials to the ROI-level results (red curves in Figure S4). A preponderance of negative responses in young subjects is particularly apparent for all right hemisphere regions and left SMA.

Finally, we checked whether any of the confounds we defined (Table S1) had significant effects. We specified an F-contrast as an identity matrix over the confound variables. This was essentially a model comparison of the GLM with versus without the confounds. There were no significant effects across the whole brain. We therefore did not include these confounds in the connectivity analyses that followed.

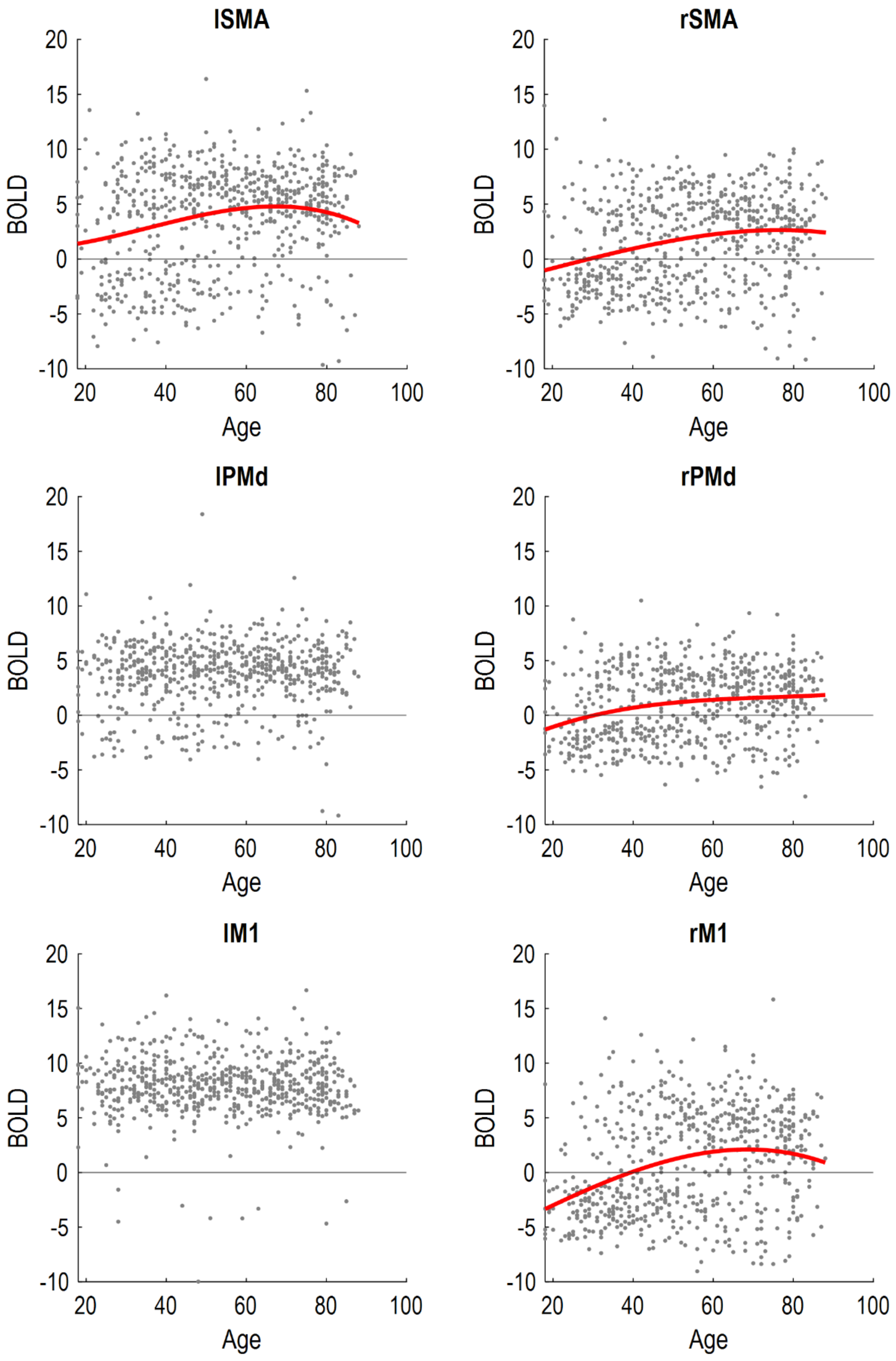

Figure S4. Correlation between age and response to Auditory+Visual trials. Non-linear curves of best fit (red lines) are plotted for those regions with a significant linear correlation (** $p < 0.05$ Bonferroni corrected).

2.4 Neural parameters

Figure S5 shows all the neural connectivity parameters from the PEB model, at the group level, following Bayesian model reduction. This procedure pruned any parameters from the model that did not contribute to the log evidence. Strikingly, there was substantially reduced driving input to lSMA (right hand plots) in negative responders, who were generally younger.

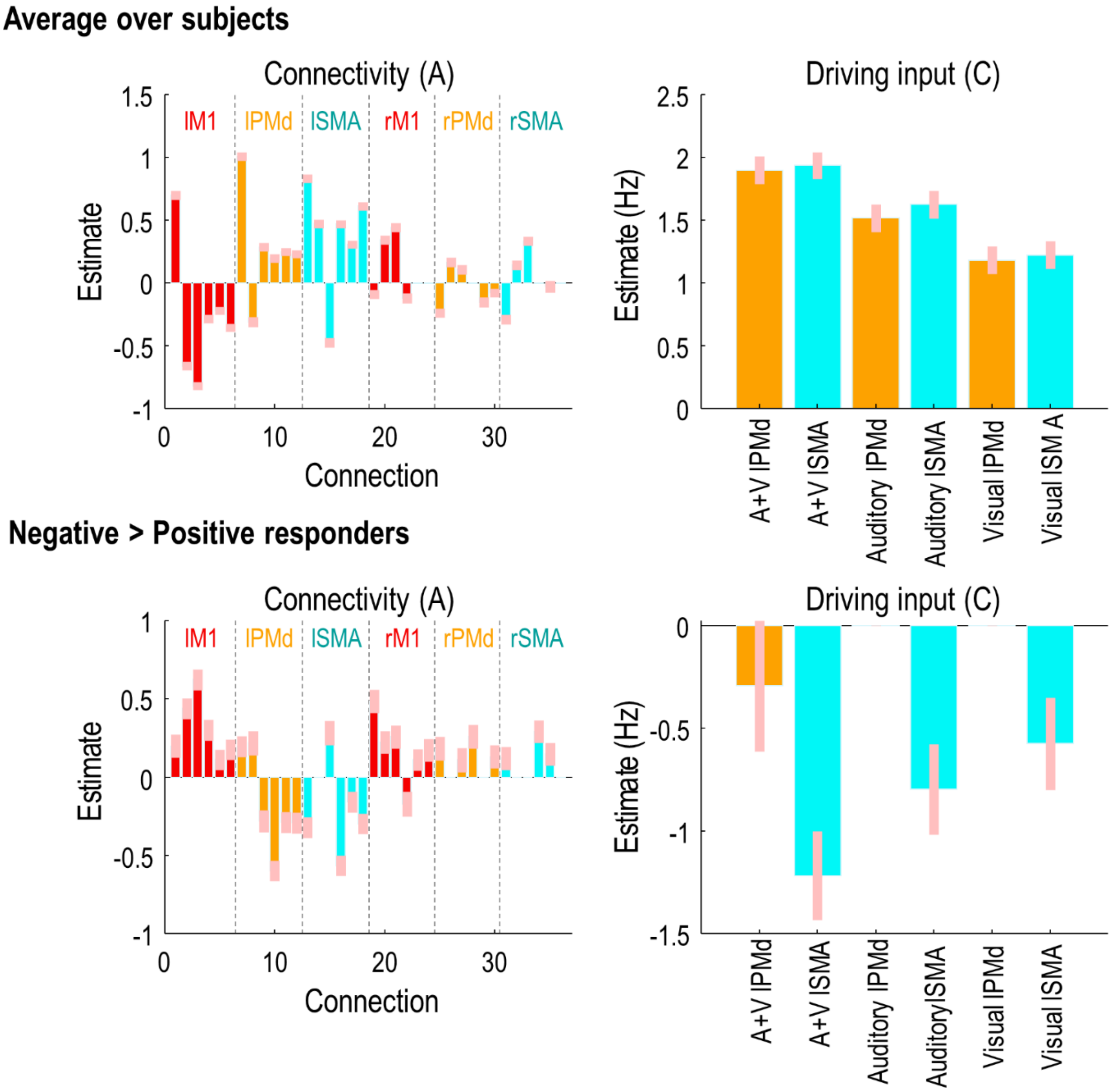

Figure S5. Posterior parameter estimates of the neural part of the model. DCM parameters were summarised at the group level using PEB. Parameters that did not contribute to the group-level log evidence (free energy) were pruned using Bayesian model reduction. Colours match Figure 1A of the main text. **Top**: the commonalities across subjects (group average). For the connectivity parameters (left), each group of six bars show the outgoing connections from the labelled region, to: (1) lM1, (2) lPMd, (3) lSMA, (4) rM1, (5) rPMd, (6) rSMA. Pink error bars are 90% Bayesian credible intervals. **Bottom:** The effect of group, where positive values indicate more positive parameter estimates for

the younger group (negative responders), whereas negative values indicate more positive values for the older group (positive responders).

2.5 Simulations in the positive responder group

We specified a DCM with the average parameters of all subjects in the positive responder group. We then varied each neural and haemodynamic parameter in turn, to determine which parameters could switch from a positive to negative BOLD response, when varied in isolation. Three parameters had this effect (Figure S6). The same three inter-hemispheric neural connections were identified in the main text as being sufficient to switch a negative responder subject to having a positive BOLD response. Whereas that analysis also identified the driving input to lPMd, this parameter could not switch from a positive to negative BOLD response.

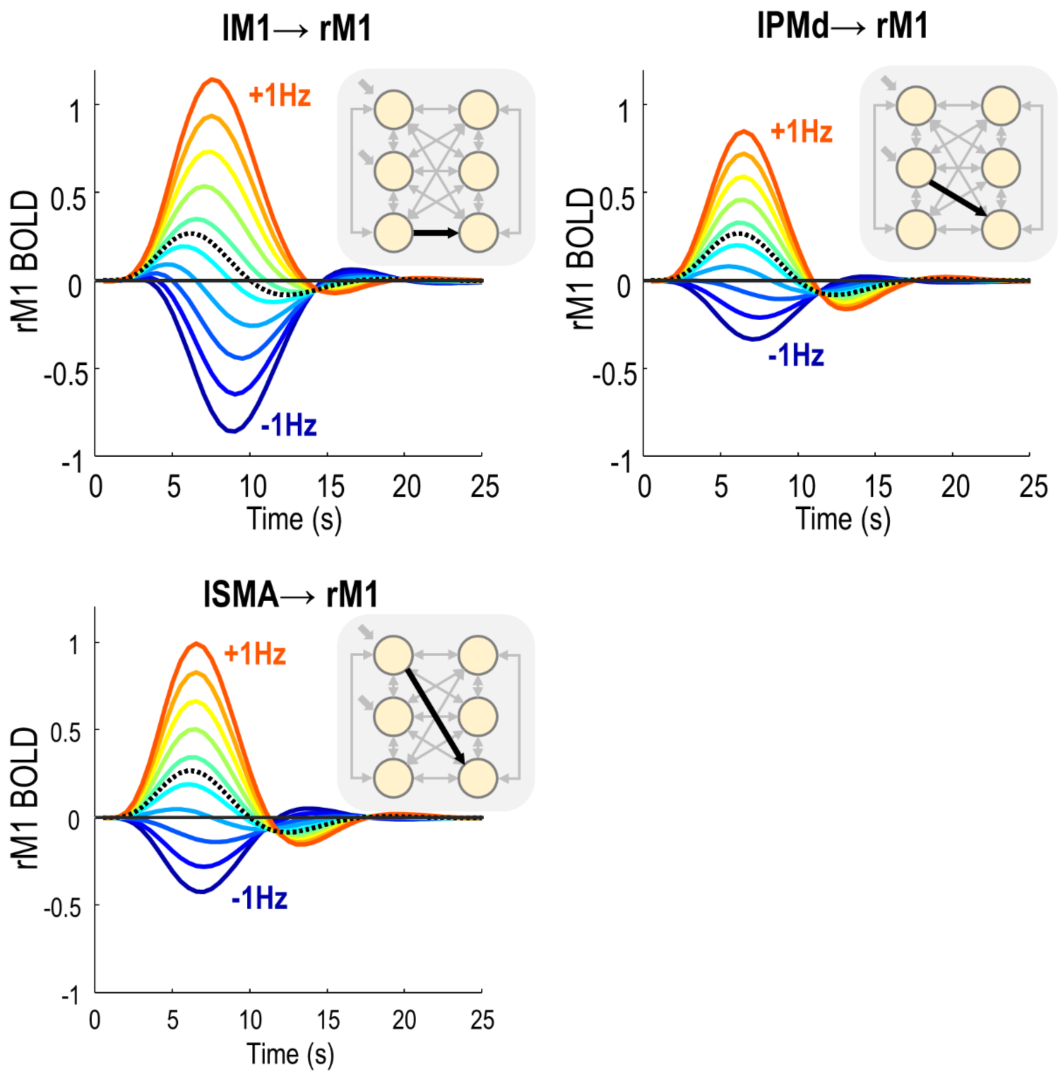

Figure S6. Simulations based on the average model parameters of positive responder subjects. Each plot shows the effect of varying one parameter (indicated by the title and the diagram, inset), on the predicted BOLD response in right M1. The black dotted line indicates the predicted BOLD response under the group average parameters for the positive responder group. The coloured lines are simulated BOLD responses, as a consequence of varying each parameter between its estimated value minus 1Hz, to its estimated value plus 1Hz.

2.6 Re-analysis with adjusted lSMA and lM1 masks

As stated in the main text, given that the left hemisphere ROIs were defined by taking the mirror image of the right hemisphere ROIs, we used the HMAT atlas to ensure that the left hemipshere ROIs were positioned in the correct anatomically defined regions. The proportions of voxels in each left hemisphere ROI were: lM1 ROI [99% M1, 1% S1]; lPMd ROI [95% PMd, 5% M1]; lSMA ROI [100% SMA]. To confirm that the slight misalignment in lM1 and lPMd with the HMAT atlas didn't alter the results, we re-ran the analyses with the lM1 ROI trimmed to remove the 1% of voxels that were labelled as S1 by the atlas, and the lPMd ROI trimmed to remove the 5% of voxels labelled as M1 by the atlas.

All results with these adjusted ROIs were qualitatively the same, with only trivial differences in the estimated connectivity parameters from the main analysis (typically less than 0.01Hz). Figure S7 shows the same connections as Figure 7 of the main text, using the altered lM1 and lPMd ROIs.

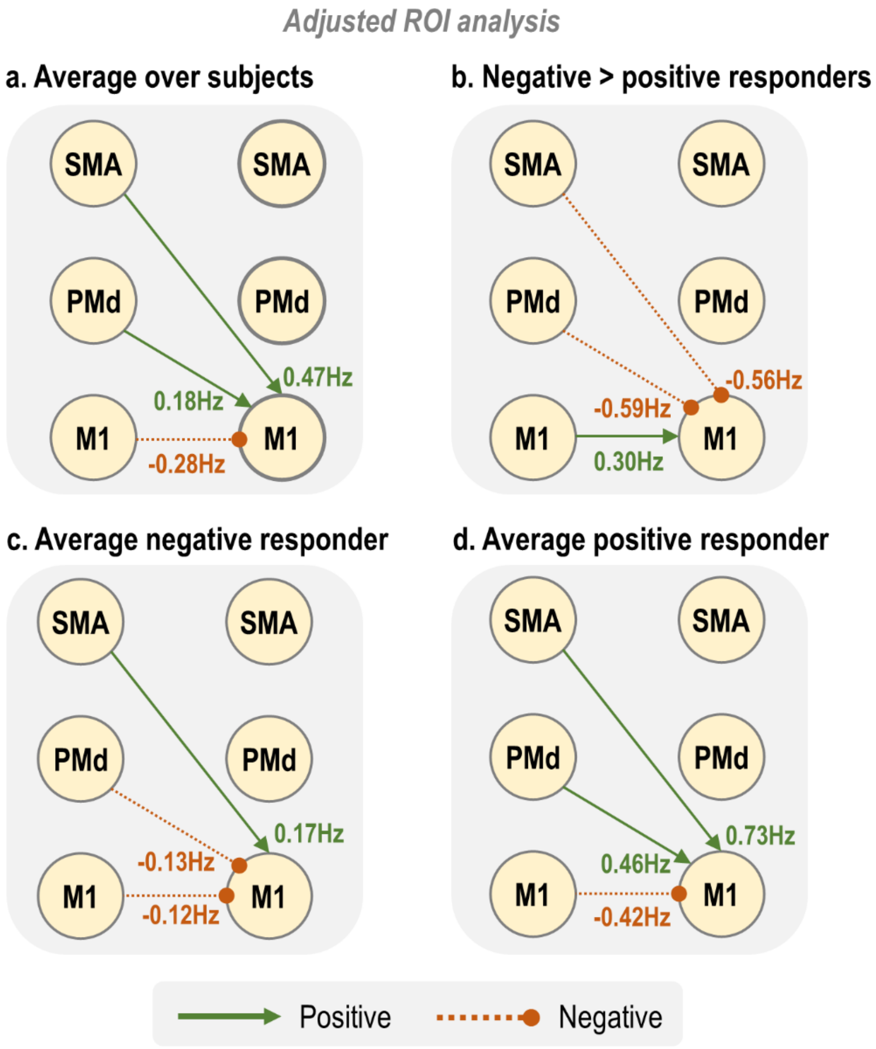

Figure S7. Summary of connections that showed a group difference and could determine the sign of the BOLD response, as demonstrated using simulations. Positive connections are excitatory, whereas negative connections are inhibitory. This figure is the uses adjusted lPMd and lM1 ROIs as described in the text.

Supplementary References

1. Xia, M., J. Wang, and Y. He, *BrainNet Viewer: a network visualization tool for human brain connectomics.* PloS one, 2013. **8**(7): p. e68910.
2. Mayka, M.A., et al., *Three-dimensional locations and boundaries of motor and premotor cortices as defined by functional brain imaging: a meta-analysis.* Neuroimage, 2006. **31**(4): p. 1453-1474.
